# Restructuring physical micro-environments to reduce the demand for meat: a systematic review and qualitative comparative analysis

**DOI:** 10.1016/S2542-5196(18)30188-8

**Published:** 2018-09

**Authors:** Filippo Bianchi, Emma Garnett, Claudia Dorsel, Paul Aveyard, Susan A Jebb

**Affiliations:** a University of Oxford, Nuffield Department of Primary Care Health Sciences, Oxford, UK; b University of Cambridge, Department of Zoology, Cambridge, UK; c Heinrich Heine University Düsseldorf, Department of Psychology, Düsseldorf, Germany

## Abstract

**Background:**

Reducing meat consumption could help to protect the natural environment and promote population health. Interventions restructuring physical micro-environments might help to change habitual behaviour. We synthesised the scientific evidence pertaining to whether, and which, interventions restructuring physical micro-environments effectively reduce the demand for meat.

**Methods:**

We did a systematic review of quantitative studies evaluating the effectiveness of interventions restructuring physical micro-environments to reduce the demand for meat. We identified relevant records by searching six electronic databases (CAB Abstracts, Embase, PsycINFO, Science Citation Index, MEDLINE, and Dissertations & Theses) on Aug 31, 2017, contacting experts, screening publicly accessible online resources, and searching references. We included studies that evaluated the effectiveness of interventions restructuring physical micro-environments to reduce the demand for meat, defined as the actual or intended consumption, purchase, or selection of meat in real or virtual environments. We extracted data pertaining to the study samples, the interventions, and meat demand at the follow-up closest to intervention completion and at the longest follow-up, with the former representing our primary outcome. We synthesised data narratively and did a qualitative comparative analysis to identify configurations of intervention characteristics associated with, and those not found to be associated with, significant reductions in meat demand. Our Systematic Review is registered with PROSPERO, number CRD42017081532.

**Results:**

Of 10 733 titles and abstracts screened for eligibility, we assessed 60 full papers and included 14 papers reporting on 18 studies with 22 intervention conditions. Three interventions reducing the portion size of meat servings reduced meat consumption in randomised trials. Three interventions providing meat alternatives with supporting educational material were associated with reduced meat demand in pre-post design studies. Three of four interventions altering the sensory properties (eg, visual presentation) of meat or meat alternatives at point of purchase reduced meat demand in randomised trials. Four interventions repositioning meat products to be less prominent at point of purchase were associated with lower meat demand, but only two such interventions reached statistical significance in a randomised trial and a multiple treatment reversal design. Only one of five interventions manipulating the description of meat or meat alternatives at point of purchase was associated with lower meat demand in a multiple treatment reversal design. Evidence from randomised trials evaluating a pricing intervention or interventions restructuring several aspects of micro-environments was too scarce or inconsistent to be conclusive. The results from our qualitative comparative analysis supported the findings of this narrative synthesis.

**Interpretation:**

Some interventions restructuring physical micro-environments could help to promote lower demand for meat. Interventions reducing portion sizes of meat servings, providing meat alternatives, or changing the sensory properties of meat and meat alternatives at point of purchase offered the most promise in the context of experimental studies.

**Funding:**

None.

## Introduction

Livestock negatively affects the environment, degrading land, polluting fresh water resources, threatening natural biodiversity, and contributing to greenhouse gas emissions that advance anthropogenic climate change.^1^, ^2^, ^3^, ^4^ The environmental changes attributable to livestock might in turn affect human global health through numerous pathways, including antimicrobial resistance and the spread of vector-borne diseases.^5^, ^6^, ^7^ Supply-side measures are important to mitigate the environmental effect of livestock,^8^, ^9^, ^10^ but research suggests that reducing the demand for meat is necessary to achieve climate change targets agreed upon by the international community.^11^, ^12^, ^13^, ^14^ Furthermore, because consumption of red and processed meat is associated with some non-communicable diseases,^15^, ^16^, ^17^, ^18^, ^19^, ^20^, ^21^, ^22^ tackling the demand for these foods provides the most direct opportunity to simultaneously protect the environment and promote population health.^23^, ^24^ However, little is known about how to promote this behaviour change.^11^, ^25^ To date, initiatives aimed at promoting environmentally sustainable lifestyles have generally focused on providing information about the effect of anthropogenic activities on the natural environment.^26^ Nevertheless, information provision alone is thought to be insufficient to “make a discernible impact on behaviour at the level needed”,^26^ and a review found that simply conveying the environmental effect of meat production did not influence meat purchases.^27^ The restricted effectiveness of interventions exclusively targeting conscious determinants of human behaviour (eg, knowledge and values) might be explained by the insight that characteristics of physical micro-environments (ie, the “settings in which people may gather for specific purposes and in which they may acquire or consume food”^28^), exert a powerful influence on behaviour and might override conscious intentions.^26^, ^29^ After learning about greenhouse gas emissions caused by livestock, one might consciously intend to eat less meat, but fail to behave accordingly when dining at a canteen that lacks appealing meat-free alternatives, or when shopping in a supermarket that offers discounts for larger portions of meat products. Dual-process models of human behaviour postulate that habitual behaviours, such as the consumption of meat in many high-income and middle-income countries, are often driven by automatic processes that are in turn influenced by features of physical micro-environments, rather than being the exclusive result of conscious and rational thought processes.^26^, ^30^, ^31^ Accordingly, these micro-environments can be designed purposefully to shape habitual behaviours, and there is growing interest in how this behavioural approach could be used to promote planetary health.^26^, ^32^ In this systematic review, we aimed to synthesise the scientific evidence pertaining to whether, and which, interventions restructuring physical micro-environments effectively reduce the demand for meat.

**Research in context**

**Evidence before this study**

Red and processed meat consumption is associated with higher risks of developing chronic conditions, and there is growing concern that meat production might also detrimentally affect human health through its impact on the natural environment. Reducing the demand for meat could help to simultaneously promote population health and mitigate the anthropogenic impact on the natural environment. However, little is known about how to promote this behaviour change and policy makers remain unable to use this opportunity to promote planetary health. Dual process models of human behaviour suggest that habitual behaviours, such as the consumption of meat in many high-income and middle-income countries, are often influenced by characteristics of the physical micro-environments in which people live and make choices. As such, these micro-environments could be purposefully designed to reduce the demand for meat. In this systematic review, we synthesised the evidence from experimental studies evaluating the effectiveness of interventions restructuring physical micro-environments to reduce the actual or intended consumption, purchase, or selection of meat. We searched six electronic databases (CAB Abstracts, Embase, PsycINFO, Science Citation Index, MEDLINE, and Dissertations & Theses: Global full-text) from database inception until the latest available date on Aug 31, 2017, using a predefined algorithm that included terms relating to the target products (eg, meat), processes of change (eg, reduction), the behaviour of interest (eg, consumption), and a filter to identify intervention studies. We identified further records by contacting experts in the field, screening references of relevant papers, and searching publicly accessible online resources. Two members of the research team (FB and EG or FB and CD) independently assessed the eligibility of all records identified, extracted prespecified data from all eligible studies, and assessed the methodological quality of these studies using the Quality Assessment Tool for Quantitative Studies.

**Added value of this study**

We considered 10 733 papers and included 14 papers reporting on 18 studies with 22 intervention conditions in our Systematic Review. Our narrative synthesis and qualitative comparative analysis suggest that interventions reducing portion sizes of meat servings, providing meat alternatives with supporting educational material, and manipulating the sensory properties of meat or meat alternatives offered the most promise to reduce meat demand. We found some evidence of effectiveness for interventions repositioning meat products to be less prominent at point of purchase. Manipulating the verbal description of meat or meat alternatives at point of purchase was not found to be an effective approach. The evidence pertaining to pricing interventions and to interventions restructuring multiple elements of micro-environments was inconclusive.

**Implications of all the available evidence**

To our knowledge, this Article provides the first systematic synthesis of the effectiveness of interventions restructuring micro-environments to reduce the demand for meat. This Article might provide preliminary evidence to inform practice of institutions wishing to reduce meat consumption to promote planetary health. However, given the paucity of evidence available to date, these findings are of more direct importance to the scientific community working towards developing evidence-based solutions for reducing population-wide meat consumption to simultaneously protect the natural environment and promote population health.

## Methods

### Search strategy and selection criteria

In this systematic review, we followed methods set out by Cochrane for conducting our searches, screening, data extraction, and data synthesis. We included any experimental intervention study, including pilot and feasibility studies, that evaluated the effectiveness of interventions restructuring physical micro-environments to reduce the demand for meat, defined as the actual or intended consumption, purchase, or selection of meat in real or virtual environments. Interventions not explicitly aimed at reducing meat demand were eligible if they altered physical micro-environments in ways that could reduce the selection of meat or encourage the uptake of meat alternatives in discrete choice situations, where the selection of meat-free options implied the rejection of meat. A study could be included if the outcome was objective or self-reported measures of meat demand. Eligible comparators were, in order of preference, no or minimal intervention controls, pre-intervention baseline, or other eligible interventions. We excluded interventions promoting general dietary patterns (eg, Mediterranean diet) and interventions not featuring any component of environmental restructuring (eg, purely educational interventions), as well as qualitative and non-experimental studies (appendix). There were no exclusion criteria pertaining to the publication status, publication year, language, length of follow-up, or population, except for people diagnosed with clinical conditions for which it is required to consume specific amounts of meat. We did searches jointly for this review and a companion review (unpublished).^33^ We searched six electronic databases (CAB Abstracts, Embase, PsycINFO, Science Citation Index, MEDLINE, and Dissertations & Theses: Global full-text) from database inception until the latest available date on Aug 31, 2017, using a predefined algorithm that included terms relating to the target products (eg, meat), processes of change (eg, reduction), the behaviour of interest (eg, consumption), and a filter to identify intervention studies (appendix). We also searched publicly accessible online resources, contacted experts in the field, and conducted iterative backward and forward reference searches for all papers included in the present and companion review.^34^ Two members of the research team (FB and EG or FB and CD) independently assessed the eligibility of all records identified, extracted prespecified data from all eligible studies, and assessed the methodological quality of these studies using the Quality Assessment Tool for Quantitative Studies.^35^, ^36^ If needed, we contacted authors to seek further information about their research. We resolved any disagreements through discussion.

This systematic review is registered with PROSPERO, number CRD42017081532.^37^

### Data synthesis

We extracted data pertaining to the sample characteristics, the interventions, and the self-reported or objective measures of meat demand. Where available, we extracted results pertaining to attitudes, subjective social norms, and perceived behavioural control of consuming, purchasing, or selecting (less) meat and results pertaining to biomarkers of health risk, including blood pressure, blood cholesterol, blood glucose, and bodyweight. When data for multiple follow-up times were available, we extracted that pertaining to the follow-up closest to intervention completion and the longest follow-up, with the former representing our primary outcome.

We synthesised results narratively and grouped them according to the nature of the intervention: reducing portion sizes of meat servings; providing meat alternatives; altering the sensory properties of meat or meat alternatives, such as changing the visual presentation or hedonic value of these products at point of purchase; repositioning meat products to reduce their prominence at point of purchase; manipulating the description or label of meat or meat alternatives; changing the price of meat; or altering multiple elements of physical micro-environments. The results of a study included in this review were based on our analysis of its raw dataset.^38^ As this dataset was not detailed enough to allow exploration of whether it met the assumptions underlying the statistical methods used, we recommend caution when interpreting the results of this individual paper.^38^ To augment our narrative synthesis, we did an exploratory crisp-set qualitative comparative analysis to identify configurations of intervention characteristics associated with, and those not found to be associated with, statistically significant reductions in the demand for meat in at least 75% of more than one evaluation. We selected a criterion p value of less than 0·05 to define whether the reduction in meat demand was statistically significant. The configuration of characteristics underlying each intervention was determined using a binary coding system to describe whether the interventions featured one or more of the strategies outlined above, whether the intervention additionally featured educational or training components, and whether the outcome was actual as opposed to virtual or intended consumption, purchase, or selection of meat. The evaluation of one intervention was excluded from qualitative comparative analysis as its description was not sufficiently detailed to allow for appropriate categorisation.^39^ Where multiple follow-up times were available, we focused on the one closest to intervention completion in our qualitative comparative analysis. Further details on qualitative comparative analysis in systematic reviews can be found in a methodological paper,^40^ which we followed to plan and conduct our analysis.

We used the software fsQCA 3.0 for Mac for our analysis.

### Role of the funding source

There was no funding source for this specific study. The corresponding author had full access to all the data in the study and had final responsibility for the decision to submit for publication.

## Results

Of 10 733 titles and abstracts screened for eligibility, we assessed 60 full papers and included 14 papers reporting on 18 studies and 22 intervention conditions in our review (figure).

**Figure:**
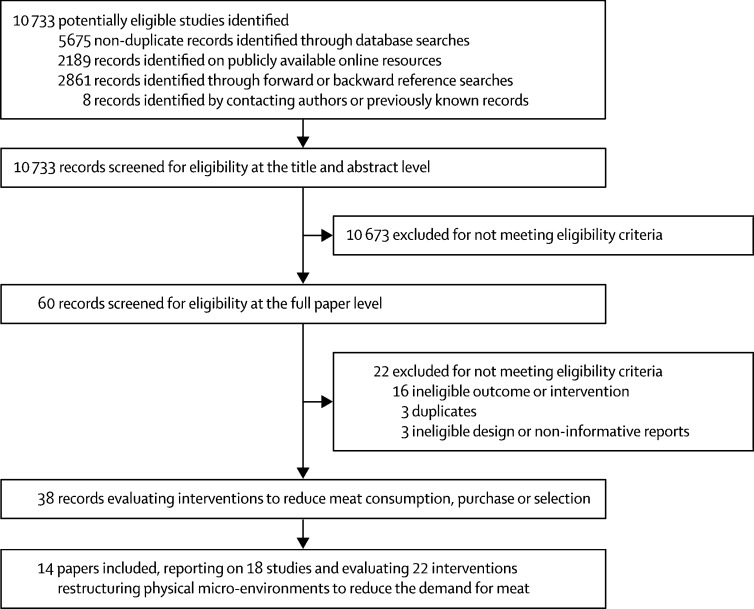
Study selection

Of the 18 studies we included, the methodological quality was strong in three, medium in 11, and low in four (table 1). 12 studies used a parallel, crossover, or factorial randomised controlled trial design, three used a multiple treatment reversal design, and three used a pre-post design. 13 studies recruited participants at the individual level, four recruited canteens or restaurants, and one recruited small businesses. All studies analysed data at the individual level or on the basis of individual food purchases. Six studies reported data on meat consumption, five reported data on meat purchases or selection, and seven reported data on meat purchases or selection in virtual settings. Additionally, four studies reported on attitudes towards eating meat and three studies reported on at least one prespecified biomarker of health risk. Our review includes 11 290 observations on individuals, individual food purchases, or individual questionnaire responses at the follow-up closest to intervention completion. Where reported, mean age ranged from 20 to 52 years (median 34) and the proportion of female participants ranged from 0% to 84% (median 53%). Of 22 interventions, three reduced the portion size of meat servings in restaurants or laboratory settings, three provided meat alternatives to free-living individuals (ie, those not being observed in a laboratory setting), four altered the visual aspects or the hedonic appeal of meat or meat alternatives, four repositioned meat products to reduce their prominence at point of purchase, five manipulated menus and meal booking systems by changing the verbal description or label of meat or meat alternatives, one used a pricing intervention, and two changed multiple elements of a university canteen or of small businesses (table 2).

**Table 1:** Study level characteristics

	**Eligibility**	**Recruitment**	Attrition and sample size*	**Publication status**	**Effective Public Health Practice Project Quality Assessment Tool for quantitative studies**†
**Randomised controlled trial**‡					
Bacon and Krpan (2018), UK^41^	Individuals had to be resident in the UK, have English as their first language, and not follow a diet precluding the choice of meat	Individuals were recruited through the Prolific Academic research platform	T1: n=564; attrition unknown§	Peer reviewed publication	Medium
Kongsbak et al (2016), Denmark^42^	Individuals had to be male university students aged between 18 and 29 years old	Individuals were recruited through advertisement on social media and on Aalborg University campus	T1: n=65, attrition 0%	Peer reviewed publication	Medium
Kunst and Hohle (2016), study 2b, Norway^43^	Individuals had to be Americans from the USA	Individuals were recruited through Amazon Mechanical Turk	T1: n=101, attrition unknown§	Peer reviewed publication	Low
Kunst and Hohle (2016), study 5, Norway^43^	NA	Individuals were recruited through Amazon Mechanical Turk	T1: n=190, attrition unknown§	Peer reviewed publication	Low
Kunst and Palacios Haugestad (2018), American sample, Norway^44^	Individuals had to be Americans from the USA who consumed meat and were 18 years or older	Individuals were recruited through Amazon Mechanical Turk	T1: n=178, attrition unknown§	Peer reviewed publication	Low
Kunst and Palacios Haugestad (2018), Ecuadorian sample, Norway^44^	Individuals had to be Ecuadorians who consumed meat and were 18 years or older	Individuals were recruited through snowball sampling on social networks	T1: n=183, attrition unknown§	Peer reviewed publication	Low
McClain et al (2013), USA^45^	Individuals had to be 18–23 years old, have a meal plan with the residence dining hall, and eat at the dining hall at least three days per week	A convenience sample was recruited by approaching students who used one of the four participating cafeterias	T1: n=525 (individual responses), attrition NA	Peer reviewed publication	Strong
Sorensen et al (2005), USA^39^	Small businesses had to be manufacturing industries with 50–150 employees, with at least 25% of workers being first generation or second generation immigrants or people of colour, a turnover rate during the past year of less than 20%, and the capacity to decide to participate	Eligible small businesses were actively approached and asked to participate	T1: n=1740 (individual responses), attrition NA (8% of worksites withdrew)	Peer reviewed publication	Medium
Vermeer et al (2010), The Netherlands^46^	Individuals had to be 18 years or older	Individuals visiting a Dutch fast food outlet were approached and asked to participate in the study after they made their purchase	T1: n=137, attrition 9%	Peer reviewed publication	Medium
**Crossover randomised controlled trial**‡					
Reinders et al (2017), The Netherlands^47^	Individual meals had to be of the relevant menu items (eg, exclusion of vegetarian meals, child menus, and special offerings), coming from parties with fewer than 12 orders, and from customers who completed questionnaires	All eligible individual orders placed during the study period in participating restaurants were recorded; restaurants were actively approached for recruitment	T1: n=1006, attrition NA	Peer reviewed publication	Strong
Rolls et al (2010), USA^48^	Individuals had to be 20–45 years old, have a BMI between 18 and 40 kg/m^2^, regularly eat three meals per day, and like and be willing to eat all three foods served in the test meals; individuals were excluded if they were dieting to gain or lose weight, had food allergies or restrictions, were taking medications known to affect appetite, or were smokers, athletes in training, pregnant or breastfeeding, had symptoms of depression, or had disordered attitudes towards food	Individuals were recruited through advertising in local newspapers and university mailing lists	T1: n=48, attrition 0%	Peer reviewed publication	Medium
**Factorial randomised controlled trial**‡					
Campbell-Arvai et al (2014), USA^49^	Undergraduate students living on campus	Individuals were actively approached and invited to take part in the experiment upon entering the dining facilities on campus	T1: n=319, attrition 0%	Peer reviewed publication	Medium
**Multiple treatment reversal**‡¶					
Stewart et al (2016), study 1, UK^38^	University dining halls with appropriate booking systems	The staff of eligible university dining halls were actively approached; all bookings placed during the study were recorded	T1: n=5280 (individual orders), attrition NA	Unpublished	Medium
Stewart et al (2016), study 2, UK^38^	University dining halls with appropriate booking systems	The staff of eligible university dining halls were actively approached; all bookings placed during the study were recorded	T1: n=782 (individual orders), attrition NA	Unpublished	Medium
Stewart et al (2016), study 3, UK^38^	University dining halls with appropriate booking systems	The staff of eligible university dining halls were actively approached; all bookings placed during the study were recorded	T1: n=61 (individual orders), attrition NA	Unpublished	Medium
**Pre-post design**‡					
Clark (2017), UK^50^	Individuals had to be aged between 21–50 years, have a BMI of 18–28 kg/m^2^, be healthy men or women (premenopausal), have good spoken and written English, consume four to five portions of red or processed meat per week, not smoke, not have a chronic disease, not be pregnant or breast feeding, not use chronic medication (excluding over the counter medication and oral contraceptives), not have participated in other research 3 months before screening, and not have clinically significant findings at screening	Individuals were recruited through advertising in newspapers, on social media pages, and in different online and offline facilities of the University of Nottingham	T1: n=26, attrition 39·5%; T2: n=22, attrition 48·8%	Unpublished	Medium
Flynn et al (2013), USA^51^	Individuals had to have access to transport to attend study activities, be willing to try new recipes, and be contactable by telephone	Individuals were recruited through advertisement in and referral from emergency food pantries	T1: n=63, attrition 26%	Peer reviewed publication	Strong
Holloway et al (2012), UK^52^	Individuals had to consume meat at least four to five times weekly, be 18–30 years old, not take regular meals in halls of residence or not live with parents or partners, be free of chronic disease, and have a BMI of 22–27 kg/m^2^	Individuals were recruited through a brief advertising presentation to around 350 students in Nottingham University	T1: n=19, attrition 27%	Unpublished	Medium

NA=not available. BMI=body-mass index.

* T1 and T2 respectively refer to the shortest and longest available post-intervention follow-up. This information refers to data underlying the analyses of meat demand.

† The Effective Public Health Practice Project Quality Assessment tool for Quantitative Studies rating is based on study design, selection bias, confounders, blinding, data collection method, withdrawal, and dropouts. Studies with more than two weak ratings in the aforementioned dimensions were assigned a low overall rating, studies with one weak rating were assigned a medium overall rating, and studies with no weak ratings were assigned a strong overall rating.

‡ The study design refers to the design underlying the main comparison reported in this review.

§ These studies used a one-off survey with an experimental component and might only have sourced data from participants who started and completed the survey. For these studies, we consider attrition to be unknown.

¶ Multiple treatment reversal designs refer to experimental studies in which intervention periods and control periods are sequentially alternated over an extended time period.

**Table 2:** Intervention effect on or association with meat demand

	**Sample characteristics and study comparison**	**Intervention**	**Outcome**	**Results**
**Provision of meat substitutes and meat-free foods**				
Clark (2017)^50^	Sample size: intervention group n=26 (shortest post-intervention follow-up), n=22 (longest post-intervention follow-up); age: median 27 years (IQR 24–32)*; female: 57%*; comparison: pre-post design	Intervention group: 12 week intervention; provision of meat substitutes, plant-based recipes, monthly motivational newsletter and emails; participants were asked to reduce consumption of red and processed meat by 50%	Red and processed meat consumption frequency (servings per week) the month before the intervention, the last intervention month, and 2 months after the intervention, assessed with a Food Frequency Questionnaire	Red and processed meat consumption was lower during the last intervention month (median 4, range <1–10) and 2 months after the intervention (average 6, range 1–14) than at baseline (median 10, range 2–20; p<0·001)
Flynn et al (2013)^51^	Sample size: intervention group n=63†; age: mean 52 years (SD 17); female: 84%; comparison: pre-post design	Intervention group: 6 week intervention; provision of 22 plant-based recipes, sufficient meat-free foods to prepare three of the 22 recipes, weekly 30 min plant-based cooking demonstrations and taster sessions, and information that consuming meat daily is not necessary for health	Purchase of meat products (US$ spent on meat per week) during the 4 weeks before intervention and the 6 months after intervention, assessed by reviewing grocery receipts	$ per week spent on meat declined from baseline (mean 16·45, SD 2·20) to after intervention (mean 7·54, SD 0·71, p<0·001)
Holloway et al (2012)^52^	Sample size: intervention group n=19; age: mean 21 years (SD 3)‡; female: 60%‡; comparison: pre-post design	Intervention group: 4 week intervention; provision of meat substitutes, 60 min information-based motivational event about vegetarianism, four face to face sessions to motivate lower meat intakes, plant-based recipes, and information about vegetarianism	Red and white meat consumption (g per day), assessed using a 7 day food diary before intervention and during the fourth week of the intervention	Red and white meat consumption was lower during the fourth week of the intervention (mean_red_≈27, mean_white_≈15) than at baseline (mean_red_≈78, p<0·001; mean_white_≈61, p<0·001)§
**Downsizing meat portions**				
Reinders et al (2017)^47^	Sample size (meal orders): intervention n=470, control: n=536; age: mean 48·6 years (SD 17·5); female: 54%; comparison: crossover, randomised controlled trial	Intervention: for 6 weeks the portion of meat (and fish) of selected meals was reduced by 12·5% and the portion of vegetables was doubled in three restaurants; control: 6 weeks of business as usual in the three restaurants	Meat consumption assessed subtracting the g of meat returned to the kitchen from the average g of meat in each of the targeted dishes	Meat consumption from the selected dishes was significantly lower during the intervention (mean 183·1, SE 2·52) than during the control period (mean 211·1, SE 2·29, p<0·001, ηp^2^=0·064)
Rolls et al (2010)^48^	Sample size: n=48; age: mean 27 years; female: 50%; comparison: crossover, randomised controlled trial	Intervention meal: in a laboratory setting, participants were served a meal in which the meat component was reduced to 243 g, the grain component was reduced to 272 g, and the vegetable component was increased to 270 g, compared with a reference meal with 281 g meat, 326 g grains, and 180 g vegetables¶	Meat consumption (in g), measured at each meal occasion weighing the meat serving before and after consumption	Meat consumption was lower during the intervention meals (mean 126·8, SD 48) than during the control meals (mean 145·4, SD 53·3, p<0·0001)
Rolls et al (2010)^48^	Sample size: n=48; age: mean 27 years; female: 50%; comparison: crossover, randomised controlled trial	Intervention meal: In a laboratory setting, participants were served a meal in which the meat component was reduced to 187 g, the grain component was reduced to 217 g, and the vegetable component was increased to 360 g, compared with a reference meal with 281 g meat, 326 g grains, and 180 g vegetables¶	Meat consumption (in g), measured at each meal occasion weighing the meat serving before and after consumption	Meat consumption was lower during the intervention meals (mean 125·2, SD 42) than during the control meals (mean 145·4, SD 53·3, p<0.0001).
**Manipulation of the sensory properties of meat or alternatives**				
Kunst and Hohle (2016), study 2b^43^	Sample size: n=101; age: mean 35 years (SD 11); female: 60%; comparison: intervention group *vs* control group, randomised controlled trial	Intervention group: participants viewed a picture of a pork roast with the pig's head; control group: participants viewed a picture of a pork roast without the pig's head	Participants indicated whether they would select a vegetarian dish instead of the pork roast on a scale from 0 (very unlikely) to 100 (very likely)	The demand for a vegetarian dish did not differ between the intervention group (mean 52·00, SE 5·56) and control group (mean 37·88, SE 5·11, p=0·065)
Kunst and Palacios Haugestad (2018), American sample^44^	Sample size: n=178; Age: mean 36 years (SD 11)‖; female: 42%‖; comparison: intervention group *vs* control group, randomised controlled trial	Intervention group: participants viewed a picture of a pork roast with the pig's head; control group: participants viewed a picture of a pork roast without the pig's head	Participants indicated whether they would select a vegetarian dish instead of the pork roast on a scale from 0 (very unlikely) to 100 (very likely)	The demand for a vegetarian dish was higher in the intervention group (mean≈56, SE≈4) than in the control group (mean≈29, SE≈4, t[176]=5·22, p<0·001)
Kunst and Palacios Haugestad (2018), Ecuadorian sample^44^	Sample size: n=183; age: mean 27 years (SD 9)**; female: 58%**; comparison: intervention group *vs* control group, randomised controlled trial	Intervention group: participants viewed a picture of a pork roast with the pig's head; control group: participants viewed a picture of a pork roast without the pig's head	Participants indicated whether they would select a vegetarian dish instead of the pork roast on a scale from 0 (very unlikely) to 100 (very likely)	The demand for a vegetarian dish was higher in the intervention group (mean≈46, SE≈4) than in the control group (mean≈33, SE≈45, t[181]=2·59, p=0·01)
Campbell-Arvai et al (2014)^49^	Sample size: factor n=160, no factor n=160; age: NA; female: 53%; comparison: factor *vs* no factor, factorial randomised controlled trial	Factor (intervention group menus): food menus including five appealing meat-free options and a range of non-vegetarian dishes; no factor (control group menus): food menus including five less appealing meat-free options and a range of non-vegetarian dishes	Simulated food choices were dichotomised in meat options *vs* meat-free options	Participants viewing intervention group menus had lower odds of selecting meat options than did those viewing control group menus (OR 0·49, 95% CI 0·36–0·66)
**Repositioning of meat**				
Kongsbak et al (2016)^42^	Sample size: intervention group n=33, control group: n=32; age: mean 24 years; female: 0%; comparison: intervention group *vs* control group, randomised controlled trial	Intervention group: participants served themselves ad libitum from a buffet including, in order of appearance: standard size plates, salad components served in separate bowls, dressings, pasta, bread, and meatballs; control group: participants served themselves ad libitum from a buffet including, in order of appearance: standard size plates, pasta, bread, meatballs, mixed salad (ie, all the salad components served together), and dressings	Selection of meatballs (in g) assessed using radio frequency identification technologies of the intelligent buffet	Selection of meatballs did not differ significantly between the control group (mean 194·6, SD 78·6) and the intervention group (mean 156·2, SD 71·1; p=0·078), after adjusting for BMI, age, and selection of salad, pasta, and bread
Campbell-Arvai et al (2014)^49^	Sample size: factor n=160, no factor n=160; age: NA; female: 53%; comparison: factor *vs* no factor, factorial randomised controlled trial	Factor (intervention group menus): food menus from which the meat options were removed and repositioned on a board 3·5 m away; no factor (control group menus): food menus containing a range of meat-free and meat-based options	Simulated food choices were dichotomised in meat options *vs* meat-free options	Participants viewing intervention group menus had lower odds of selecting meat options than did those viewing control group menus (OR 0·24, 95% CI 0·18–0·36)
Stewart et al (2016), study 2^38^	Sample size: orders during the intervention period n=384 (227 meat orders, 157 meat-free orders); orders during the control period n=398 (346 meat orders, 52 meat-free orders); age: NA; female: NA; comparison: multiple treatment reversal	Intervention period: meat options appeared after meat-free options in two university online meal booking systems over 3 observation weeks; control period: meat options appeared before meat-free options in two university online meal booking systems over 3 observation weeks	Number of meat-containing meals (including fish) and meat-free meals purchased	Adjusted for college site, meal purchases over the intervention period had 0·12 times the odds of containing meat compared with meals purchased during the control period (OR 0·12, 95% CI 0·08–0·18; p<0·001)††; the likelihood of selecting a meat option was significantly higher in one of the two college sites at which the intervention was tested
Stewart et al (2016), study 3^38^	Sample size: orders during the intervention period n=31 (26 meat orders, five meat-free orders); orders during the control period n=35 (30 meat orders, five meat-free orders); age: NA; female: NA; comparison: multiple treatment reversal	Intervention period: for 2 weeks meat-free options were repositioned to be the default option in a university online meal booking system; students not actively changing their selection to the meat option were served a plant-based meal; control period: for 2 weeks meat options were left as the default option in a university online meal booking system; students not actively changing their selection to vegetarian were served meat	Number of meat-containing meals (including fish) and meat-free meals purchased	Meal purchases over the intervention period had 0·87 times the odds of containing meat compared with meals purchased over the control period, but this effect did not reach statistical significance (OR 0·87, 95% CI 0·23–3·33, p=0·87)††
**Manipulating the description or labelling of meat or alternatives**				
Bacon and Krpan (2018)^41^	Sample size: intervention group n=185, control group n=194; age: mean 36 years; female: 51%; comparison: intervention group *vs* control group, randomised controlled trial	Intervention group: food menu containing three meat and five meat-free options, in which the description of the first meat-free dish was changed from “Risotto Primavera” to “Fresh Seasonal Risotto Primavera”; control group: food menu containing three meat and five meat-free options	Simulated food choices were dichotomised into meat options (chicken cacciatora, steak frites, or hamburger) *vs* meat-free options	The odds of selecting a meat option did not differ between the intervention group and the control group (OR 1·1, p=0·677)
Bacon and Krpan (2018)^41^	Sample size: intervention group n=185, control group: n=194; age: mean 35 years; female: 52%; comparison: intervention group *vs* control group, randomised controlled trial	Intervention group: food menu that contained three meat and five meat-free options, in which the first meat-free dish (ie, “Risotto Primavera”) was highlighted as the “Chef's recommendation”; control group: food menu containing three meat and five meat-free options	Simulated food choices were dichotomised into meat options (chicken cacciatora, steak frites, or hamburger) *vs* meat-free options	The odds of selecting a meat-based meal did not differ between intervention group and control group (OR 1·37, p=0·180)
Kunst and Hohle (2016), study 5^43^	Sample size: n=190; age: mean 34 years (SD 10); female: 52%; comparison: intervention group *vs* control group, randomised controlled trial	Intervention group: food menu with eight meat-based meals, which were described as “cow” and “pig” options; control group: food menu with eight meat-based meals, which were described as “beef” and “pork” options	Participants indicated whether they would select a meat-free meal instead of the meat options on a scale from 0 (very unlikely) to 100 (very likely)	The demand for meat-free meals did not differ between the intervention group (mean 43·12, SE 3·84) and the control group (mean 33·78, SE 3·49, p=0·074)
Campbell-Arvai et al (2014)^49^	Sample size: factor n=160, no factor n=160; age: NA; female: 53%; comparison: factor *vs* no factor, factorial randomised controlled trial	Factor (intervention group menus): food menus containing a range of meat-based options and meat-free options that were differentiated with a leaf symbol indicating that eating less meat can help reduce our environmental impact; no factor (control groups menus): food menus containing a range of meat-free and meat-based options	Simulated food choices were dichotomised into meat options *vs* meat-free options.	The odds of selecting a meat-based dish did not differ between participants viewing the intervention group or the control group menus (OR 0·92, 95% CI 0·69–1·2)
Stewart et al (2016), study 1^38^	Sample size: orders during intervention period n=2784 (2373 meat orders, 411 meat-free orders); orders during control period n=2496 (2177 meat orders, 319 meat-free orders); age: NA; female: NA; comparison: multiple treatment reversal	Intervention group period: meat options were labelled as “meat” instead of “standard” or “normal” in four university online meal booking systems over 12 observation weeks; control group period: meat options were labelled as “standard” or “normal” in four university online booking systems over 12 observation weeks	Number of meat-containing meals (including fish) and meat-free meals purchased	Adjusted for college site, meal purchases over the intervention group period had 0·83 times the odds of containing meat compared with meals purchased over the control group period (OR 0·83, 95% CI 0·71–0·98, p=0·02)††; the likelihood of selecting a meat option was significantly higher in some colleges compared with others
**Pricing**				
Vermeer et al (2010)^46^	Sample size: n=137; age: mean 25 years (SD 10); female: 66%; comparison: intervention group *vs* control group, randomised controlled trials	Intervention group: three portions of chicken nuggets were priced with a proportional system—€2·35 for a small portion, €3·50 for a medium portion, and €5·80 for a large portion; control group: three portions of chicken nuggets were priced with a value system—€2·75 for a small portion, €3·50 for a medium portion, and €5·00 for a large portion	Simulated selection of small, medium, or large portion of nuggets was dichotomised in small *vs* other and in large *vs* other	Authors found no effect of pricing on the selection of different portion sizes among the general population
**Multicomponent changes to the micro-environment**				
McClain et al (2013)^45^	Dining halls: intervention group n=2, control group n=2; questionnaire responses: intervention group n=247; control group n=278‡‡; age: 20 years; female: 53%; comparison: intervention group *vs* control group, randomised controlled trial	Intervention group: 4 week marketing campaign featuring flyers, labels, healthy choice indicators of meat-free foods, and sample meat-free dishes at the entrance of the canteen; control group: 4 weeks of business as usual	Consumption frequency of high-fat meats (in servings per week) assessed at the baseline and directly after the intervention with a food frequency questionnaire	In the control group high-fat meat intake increased by 0·9 servings per week, while it decreased by 0·9 servings per week in the intervention group (time × condition interaction: p=0·04)
Sorensen et al (2005)^39^	Small businesses: intervention group n=13, control group n=13; questionnaire responses: intervention group n=807, control group n=933‡‡; age (adjusted for worksite clustering): intervention group 44 years, control group 43 years; female: 33%; comparison: intervention group *vs* control group, randomised controlled trial	Intervention group: 18 month multicomponent intervention to reduce red meat intake and smoking and to increase physical activity, fruit, vegetable, and multivitamin intake; specific interventions were designed within each worksite under the advice of a hygienist, and included policies aimed at offering healthful food options at company meetings, system oriented interventions, interactive activities, and education; control group: smoking cessation services	Consumption frequency of red meat (in servings per week) assessed with a food frequency questionnaire at baseline and directly after intervention; responses were dichotomised in ≤3 servings per week *vs* >3 servings per week	The change in percentage of participants eating ≤3 servings per week of red meat did not differ between the intervention group (+4·1%) and control group (+3%) after adjusting for worksite clustering (p=0·72)

≈ indicates results were read from figures or graphs. NA=not available. OR=odds ratio. BMI=body-mass index.

* Baseline characteristics of the 37 participants completing some secondary outcomes extracted from the doctoral thesis on which the study was based.

† Only 60 participants provided a complete set of grocery receipts at both timepoints.

‡ Of the 25 participants recruited at baseline.

§ Results were based on an independent sample *t* test, while a dependent sample *t* test should be used for pre-post designs.

¶ Both control and intervention meals were served to each participant on two different occasions varying the energy content of the vegetable component. For the aim of this review participants' average consumption was defined as their average consumption across the two energy-varied meals.

‖ Of the 201 participants enrolled.

** Of the 202 participants enrolled.

†† A logistic regression analysis was done of the basis of raw data available from the unpublished report.

‡‡ Questionnaires were not always completed by the same individuals at baseline and at follow-up.

Two crossover randomised controlled trials found that all three interventions reducing the portion size of meat servings significantly reduced meat consumption in a real restaurant setting^47^ and a laboratory setting.^48^ In the laboratory study, reducing the portion size of meat servings by 13·5% or 33·5% led to lower meat intakes compared with a reference meal containing 281 g of meat, but participants' meat consumption did not differ between the two intervention meals.^48^ Across all meals served as part of this study, participants' average meat intake never reached the maximum amount of meat served.

In three pre-post intervention studies, all three interventions providing meat alternatives were associated with significant reductions in meat purchases or consumption.^50^, ^51^, ^52^ Two such interventions provided meat-free or meat-reduced alternatives, such as mycoprotein products, to replace meat products for 4 or 12 weeks,^50^, ^52^ and the third intervention provided more general plant-based foods as part of a 6-week plant-based cooking demonstration programme.^51^ All three interventions additionally included motivational, educational, and training components to encourage reductions in the demand for meat.^50^, ^51^, ^52^ In two studies with prolonged follow-up, there was some evidence to suggest that several months after the supply of plant-based alternatives had stopped, demand for meat remained lower than at the baseline.^51^, ^52^

Four randomised controlled trials (one of which was factorial) suggested that three of four interventions manipulating the sensory properties of meat or meat alternatives significantly reduced the demand for meat in virtual food choices. Replacing the vegetarian items on a food menu with alternative vegetarian items previously rated as more appealing by people other than study participants significantly reduced participants' demand for meat.^49^ Manipulating the visual properties of an image of a pork roast to also display the animal's head led to greater demand for plant-based alternatives in two of three randomised controlled trials evaluating this intervention.^43^, ^44^

Four studies (one randomised controlled trial, one factorial randomised controlled trial, and two multiple treatment reversal trials) evaluated four interventions that repositioned meat products to decrease their prominence at point of purchase. Two such interventions reduced or were associated with reductions in meat demand in a multiple treatment reversal study^38^ and a factorial randomised controlled trial.^49^ These interventions repositioned meat options to appear after, rather than before, vegetarian options in online meal booking systems (ie, online platforms typically used to allow students to select different meal options in university canteens^38^), or repositioned meat options from standard food menus onto a board 3·5 m away from participants in a simulated canteen setting.^49^ Two further interventions displaying vegetarian options as the default option of an online meal booking system in a multiple treatment reversal study^38^ or repositioning a meat product from the middle to the end of a buffet aisle in a randomised controlled trial^42^ were associated with reductions in meat demand, but did not reach statistically significant effects.

Four studies (two randomised controlled trials, one factorial randomised controlled trial, and one multiple treatment reversal study) evaluated five interventions manipulating food menus or meal booking systems to encourage meat-free purchases by changing the verbal description or label of meat or meat alternatives, without changing the actual sensory properties of these products. One intervention altering university meal booking systems to refer to meat options as “meat” rather than “standard” or “normal” was associated with reduced meat purchases in a multiple treatment reversal study.^38^ Conversely, interventions manipulating virtual food menus to enhance the verbal description of meat-free options,^41^ labelling vegetarian options as environmentally sustainable,^49^ or highlighting the animal origin of meat products by referring to “beef and pork dishes” as “cow and pig dishes”^43^ were not found to reduce meat demand in randomised trials.

One randomised controlled trial found no evidence to suggest that changing the price structure of three different portions of chicken nuggets (small, medium, and large) from a value pricing system (ie, decreasing price per unit with increasing portion size) to a proportional system (ie, stable price per unit across portion sizes) effectively promoted purchases of smaller portions in a simulated food choice task.^46^

Two randomised controlled trials assessed two interventions restructuring several elements of the physical micro-environment.^39^, ^45^ A marketing campaign in university canteens, featuring examples of meat-free dishes at the canteen entrance, indicators of healthy meat-free options, and educational flyers, reduced meat consumption.^45^ Conversely, there was no evidence that an 18-month multicomponent intervention targeting red meat consumption and other health behaviours reduced meat consumption in small businesses.^39^ In this intervention, staff of the participating worksites collaborated with an expert to plan individual level and environmental level interventions to promote lower meat intake and other health behaviours. Examples included policies aimed at offering healthful food options at company meetings and events,^39^ but the specific changes to the physical micro-environment targeting red meat were not reported in detail, precluding more detailed analyses of this intervention.

We included 21 intervention conditions in our qualitative comparative analysis. Three configurations of intervention characteristics were associated with significant reductions in meat demand among at least 75% of three or more evaluations (panel 1). These configurations cover 69% of the 13 interventions associated with significant reductions in meat demand.

Panel 1 **Configuration of intervention components associated with significant reductions in meat demand**

**Provision of meat alternatives and education (raw coverage: 23%, internal consistency: 100%)**

*Outcome:*

• Reduction in actual consumption, purchase, or selection of meat

*In the presence of:*

• Provision of meat alternatives
• Education or training components

*In the absence of:*

• Reducing portion sizes of meat servings
• Manipulating the description or label of meat or alternatives
• Manipulating the sensory properties of meat or alternatives
• Repositioning meat products
• Pricing
• Multiple changes to the physical micro-environment

**Reduction in portion sizes of meat servings (raw coverage: 23%, internal consistency: 100%)**

*Outcome:*

• Reduction in actual consumption, purchase, or selection of meat

*In the presence of:*

• Reducing portion sizes of meat servings

*In the absence of:*

• Provision of meat alternatives
• Manipulating the description or label of meat or alternatives
• Manipulating the sensory properties of meat or alternatives
• Repositioning meat products
• Pricing
• Multiple changes to the physical micro-environment
• Education or training components

**Manipulating the sensory properties of meat or alternatives (raw coverage: 23%, internal consistency: 75%)**

*Outcome:*

• Reduction in the purchase or selection of meat in virtual settings

*In the presence of:*

• Manipulating the sensory properties of meat or alternatives

*In the absence of:*

• Provision of meat alternatives
• Reducing portion sizes of meat servings
• Manipulating the description or label of meat or alternatives
• Repositioning meat products
• Pricing
• Multiple changes to the physical micro-environment
• Education or training components

Overall solution coverage was 69% (ie, 69% of all interventions associated with significant reductions in meat demand are covered by one of the intervention configurations above). Overall solution consistency was 90% (ie, 90% of all interventions covered by the configurations above were associated with significant reductions in meat demand). Raw coverage refers to the percentage of all interventions associated with significant reductions in meat demand that are covered by a specific intervention configuration. Internal consistency refers to the percentage of the interventions within a given configuration that were associated with reductions in meat demand.

Conversely, there was consistently no evidence of an effect for interventions manipulating the description or labelling of meat or meat alternatives at point of purchase in reducing the purchase or selection of meat in virtual settings. This configuration is reported in panel 2 and covered 38% of the eight interventions that were not found to be associated with reduced meat demand.

Panel 2 **Configuration of intervention components not found to be associated with significant reductions in meat demand**

**Manipulating the description or labelling of meat or alternatives (raw coverage: 38%, internal consistency: 100%)**

*Outcome:*

• Reduction in the purchase or selection of meat in virtual settings

*In the presence of:*

• Manipulating the description or labelling of meat or alternatives

*In the absence of:*

• Provision of meat alternatives
• Reducing the portion size of meat servings
• Manipulation of the sensory properties of meat or alternatives
• Repositioning meat products
• Pricing
• Multiple changes to the physical micro-environment
• Education or training components

Overall solution coverage was 38% (ie, 38% of all interventions that were not found to be associated with significant reductions in meat demand are covered by the intervention configuration above). Overall solution consistency was 100% (ie, all interventions covered by the configuration above were not found to be associated with significant reductions in meat demand). Raw coverage refers to the percentage of all interventions not found to be associated with significant reductions in meat demand that are covered by the intervention configuration above. As there is only one such intervention this number is identical to the overall solution coverage. Internal consistency refers to the percentage of interventions within the configuration above that were not found to be associated with reductions in meat demand.

The results of our qualitative comparative analyses were in line with the narrative synthesis suggesting that interventions reducing the portion size of meat servings, providing meat alternatives with supporting educational material, or manipulating the sensory properties of meat or meat alternatives were associated with reduced meat demand, and there was consistently no evidence of an effect for interventions only manipulating the verbal description or the label of meat or meat alternatives at point of purchase in fostering a reduction in the purchase or selection of meat in virtual settings.

Three randomised controlled trials evaluated how four interventions highlighting the animal origin of meat products influenced attitudes towards eating meat.^43^, ^44^ Of these interventions, three negatively affected attitudes towards consuming meat by referring to “beef and pork dishes” as “cow and pig dishes” on a food menu and by manipulating an image of a pork roast to display the pig's head.^43^, ^44^ The latter intervention showed worsened attitudes towards eating meat in two of three evaluations, but was not found to influence attitudes in a study including Ecuadorian participants only.^43^ No study reported data on whether the interventions enhanced participants' perceived ability to lower their demand for meat products or whether interventions influenced participants' perceived social norms of consuming, purchasing, or selecting meat (appendix).

Evidence from two pre-post design intervention studies suggested that interventions providing meat alternatives were associated with the following beneficial changes in biomarkers of health risks: a reduction in triglycerides, total cholesterol, and low-density lipoprotein cholesterol, with no change in high-density lipoprotein cholesterol following 4 weeks of meat alternatives provision,^52^ and a reduction in low-density lipoprotein cholesterol with no change in other lipid fractions or blood pressure following 3 months of meat alternatives provision.^50^ We found no evidence to suggest that any of the three interventions providing meat alternatives significantly influenced weight^50^, ^51^, ^52^ or blood pressure (appendix).^50^

## Discussion

Our systematic review found evidence to suggest that some interventions restructuring physical micro-environments can help to reduce the demand for meat. In two crossover randomised controlled trials, all three interventions reducing meat portion sizes reduced meat consumption,^47^, ^48^ and in three pre-post design studies all three interventions providing meat-free alternatives were associated with reductions in meat demand,^50^, ^51^, ^52^ with some evidence of a sustained effect.^50^, ^51^ Three of four interventions manipulating the sensory properties of meat or meat alternatives reduced meat demand in randomised trials^43^, ^44^, ^49^ and two of four interventions repositioning meat products to reduce their prominence at point of purchase led to, or were associated with, significant reductions in meat demand in a factorial randomised controlled trial and a multiple treatment reversal study.^38^, ^42^, ^49^ However, only one of five interventions manipulating the verbal description of meat or meat alternatives at point of purchase was associated with reduced demand for meat in a multiple treatment reversal design.^38^, ^41^, ^43^, ^49^ One pricing intervention evaluated in a virtual environment was not found to influence meat purchases in a randomised controlled trial.^46^ One of two interventions manipulating multiple elements of physical micro-environments effectively reduced meat consumption in a randomised controlled trial.^39^, ^45^ Interventions manipulating the sensory properties or description of meat products to highlight their animal origin negatively affected attitudes towards meat consumption in three of four randomised trials.^43^, ^44^ We found some evidence from pre-post design studies to suggest that providing meat alternatives was associated with improved blood lipid profiles^50^, ^51^ but there was no evidence that such interventions were associated with weight loss or changes in blood pressure.^50^, ^51^, ^52^

We used gold standard methods to minimise bias and comprehensively synthesise the effectiveness of interventions restructuring physical micro-environments to reduce meat demand. We did extensive searches to identify all relevant records and included unpublished manuscripts and studies not primarily focused on reducing meat demand to decrease the risk of publication bias. Additionally, we used crisp-set qualitative comparative analysis—a novel methodological technique within systematic reviews—to identify configurations of intervention characteristics associated with, and those not found to be associated with, significant reductions in the demand for meat. Nevertheless, some methodological limitations should be considered when interpreting the results of our review. Considering the novelty of this field, we decided to review all relevant interventions, regardless of the design or methodological quality of the study. This decision allowed us to produce a comprehensive synthesis of the existing evidence and reduced the risk of publication bias, but increased the likelihood of reviewing studies with weaker methodological quality. As we included non-randomised designs, it was not always possible to make direct causal inferences on the effectiveness of interventions. Some studies were not powered to detect statistically significant changes in meat demand and their results should be interpreted with caution. Most studies were implemented in high-income countries, limiting the generalisability and applicability of our results to these settings. Outcome measures often relied on self-reported data or approximated estimates, which might have introduced bias and error variance. Additionally, selection of meat products in virtual settings is a suboptimal measure of meat demand in real-life settings and might thus lack external validity.^53^, ^54^ Part of our synthesis was based on results presented in conference abstracts,^50^ dissertations,^41^, ^52^ or online reports^38^ and their conclusions could vary following further analyses and peer review. In our analysis of one study,^38^ we found that positioning meat after vegetarian options in online meal booking systems was associated with lower selection of meat, but anecdotal evidence collected by the original author suggested that many individuals involved in this study later asked to change their selection to meat. Future research should investigate how to encourage people that were cued into selecting plant-based options to pursue this dietary choice. We used our explorative qualitative comparative analysis to descriptively identify intervention characteristics associated with reduced meat demand, but these results should not be interpreted to make causal inferences about the effectiveness of interventions. Additionally, our qualitative comparative analysis did not consider the different size, design, and quality of the studies included. Finally, although using the Quality Assessment Tool for Quantitative Studies to assess the methodological quality of all eligible studies enabled us to consider studies that had various designs, we discourage readers from directly comparing the quality rating across different study designs.

The results of our review are largely in line with previous research on the effectiveness of behavioural interventions aimed at promoting environmentally sustainable or healthy behaviours.^26^ Similar to our findings on portion sizes, a systematic review^55^ concluded that reducing portion sizes might “contribute to meaningful reductions in the quantities of food…people select and consume”. However, despite the effectiveness of this strategy, reducing the portion size of meat servings in a restaurant setting was found to decrease customers' satisfaction with the meat dish, raising questions about the acceptability of this strategy for food providers aiming to maintain their customer base.^47^ A meta-analysis^56^ suggested that positioning of food products influences purchasing behaviour, and interventions repositioning meat products to be less prominent at point of purchase were consistently associated with lower meat demand, although only some reached statistical significance. The results of interventions providing meat alternatives were consistent with previous research indicating that interventions involving provision of specific foods effectively changed other eating behaviours.^57^, ^58^ The growing range of meat substitutes^59^ might therefore bring new opportunities for interventions aimed at reducing meat demand through promotion of comparable alternatives. Preliminary evidence suggests that replacing meat with these foods might also be associated with reduced cardiovascular risk factors, but the studies on which this evidence was based were affected by methodological limitations, and more structural investigations are needed to confirm or dispute these findings. Manipulating the sensory properties of meat and meat-free products was promising for encouraging lower meat demand and was implemented through two strategies: improving the hedonic appeal of meat alternatives at point of purchase^49^ or highlighting the animal origin of a meat product by displaying the animal's head.^43^, ^44^ The effectiveness of improving the hedonic appeal of meat at point of purchase was in line with previous research on the association between the hedonic appeal of foods and purchasing intentions,^60^, ^61^ whereas the effectiveness of highlighting the animal origin of a meat product by displaying the animal's head contrasted with previous studies, which found no evidence to suggest that leading participants to reflect about the animal suffering involved in the production of meat products reduced their demand for meat.^62^ It is possible that highlighting the animal suffering involved in producing meat might offer more promise for reducing meat demand when enacted through changes to physical micro-environments than through more abstract motivational tasks. We found little evidence that altering the verbal description of meat or meat-free alternatives reduced demand for meat, which contrasted with previous research suggesting that changing the verbal description of vegetable products to enhance their perceived hedonic value influenced consumption.^63^ Finally, one study evaluating a pricing intervention in a virtual task did not find evidence to suggest that this intervention reduced the demand for meat. However, a substantial body of evidence exists to suggest that price is an important determinant of food choices, including a systematic review of randomised controlled trials in grocery stores, in which economic interventions were found to be the most promising approach to change food purchasing behaviour.^54^ Further research exploring the effectiveness of pricing strategies to reduce the demand for meat is therefore warranted.

We sought to identify interventions that might promote lower meat demand at scale and, by focusing on approaches where the effectiveness is largely independent from recipients' literacy, overcome some of the social inequities that might be perpetuated by educational interventions, whose effectiveness in promoting desirable behaviour changes is more apparent among recipients with higher literacy.^30^, ^64^ In a companion review (unpublished),^33^ we showed that interventions exclusively providing information to motivate lower meat intake appeared to reduce intended, but not actual, demand for meat, and interventions restructuring physical micro-environments could help to complement educational approaches and contribute towards bridging the intention–behaviour gap. However, we argue that educational and motivational interventions remain an important part of a portfolio of strategies to reduce population-wide meat demand, as these approaches are generally feasible and acceptable^39^, ^50^ and might enhance the public's support for structural interventions to reduce the demand for meat.^7^, ^21^, ^22^

In summary, interventions restructuring physical micro-environments could help reduce the demand for meat. Reducing portion sizes of meat, providing meat alternatives with supporting educational material, and manipulating the sensory properties of meat or meat-free alternatives appeared to be promising interventions to reduce meat demand in the context of experimental studies. We found some evidence of effectiveness of interventions that repositioned meat products to reduce their prominence at point of purchase. Manipulating the verbal description of meat or meat-free alternatives on food menus or meal booking systems, without changing the sensory properties of these products, offered less promise. We found very little evidence pertaining to the effect of pricing or restructuring multiple other elements of micro-environments. The current evidence for the effectiveness of interventions restructuring physical micro-environments to reduce the demand for meat is scarce and affected by methodological limitations. Rigorous evaluation of interventions that restructure physical micro-environments to reduce meat demand should be a priority for future research aimed at providing evidence-based solutions to planetary health challenges.

For an **Abstract based on this unpublished thesis by M Clark** see https://www.cambridge.org/core/journals/proceedings-of-the-nutrition-society/article/impact-of-dietary-meat-intake-reduction-on-haematological-parameters-in-healthy-adults/7B057AB3A3AC35C56753EBD8CF48EE79
