# Supplementary material for: Restructuring physical micro-environments to reduce the demand for meat: a systematic review and qualitative comparative analysis
Source: Lancet Planet Health. 2018 Sep;2(9):e384–97. doi: 10.1016/S2542-5196(18)30188-8 (PMC6120131; doi:10.1016/S2542-5196(18)30188-8)
Supplement: Supplementary appendix [file mmc1.pdf]

THE LANCET
Planetary Health

Supplementary appendix

This appendix formed part of the original submission and has been peer reviewed. We post it as supplied by the authors.

Supplement to: Bianchi F, Garnett E, Dorsel C, Aveyard P, Jebb SA. Restructuring physical micro-environments to reduce the demand for meat: a systematic review and qualitative comparative analysis. *Lancet Planet Health* 2018; **2:** e384–97.

Restructuring physical micro-environments to reduce the demand for meat: a systematic review and qualitative comparative analysis

Filippo Bianchi, Emma Garnett, Claudia Dorsel, Paul Aveyard, Susan A Jebb

Appendix (1-4)

	Inclusion criteria	Exclusion criteria
**Population**	All were eligible.	People diagnosed with clinical conditions for which it is required to consume specific amounts of meat.
**Intervention**	Interventions restructuring one or more elements of physical micro-environments (e.g. availability, portion size, position, label, sensory properties, or price) to reduce the demand for meat with or without additional educational, motivational, or training components.	Interventions promoting general dietary patterns (e.g. Mediterranean diet) and interventions not featuring any component of environmental restructuring (e.g. purely educational interventions).
**Comparator**	In order of preference (1) no- or minimal-intervention controls, (2) pre-intervention baselines, or (3) other eligible interventions.	N/A
**Outcome**	Objective or self-reported measures of meat demand, defined as the actual or intended consumption, purchase, or selection of meat in real or virtual environments.	N/A
**Study Design**	Any experimental intervention study, including pilot and feasibility studies regardless of publication status, publication year, language, or length of follow-up.	Qualitative and non-experimental studies.

Appendix 1: Eligibility Criteria

Block	Keywords (MEDLINE)	Hits (MEDLINE)
1	((meat* or beef or veal or lamb or pork or poultry or chicken or turkey or venison or offal* or sausage* or ham or pastrami or bacon or salami or nugget* or burger* or hamburger* or black pudding* or haggis or plant-based or vegetable-based or vegetar* or vegan* or flexitarian* or ((green or environment* or sustainab*) adj2 (eat or eating or diet*))) adj7 (chang* or increas* or decreas* or improv* or reduc* or low or moderat* or shift* or eliminat* or replac* or substitut* or alternat* or cut* or more or less or free or sustainab* or promot* or encourag* or enhance* or support* or motivat* or achiev*) adj7 (consum* or intak* or eat* or purchas* or choos* or select* or prefer* or demand* or buy* or avoid* or choice* or nudg* or cook* or prepar* or cater* or behav* or inten* or attitud* or social* norm*)).ti,ab.	6781
2	((meat* or beef or veal or lamb or pork or poultry or chicken or turkey or venison or offal* or sausage* or ham or pastrami or bacon or salami or nugget* or burger* or hamburger* or black pudding* or haggis or plant-based or vegetable-based or vegetar* or vegan* or flexitarian* or ((green or environment* or sustainab*) adj2 (eat or eating or diet*))) adj7 (consum* or intak* or eat* or purchas* or choos* or select* or prefer* or demand* or buy* or avoid* or choice* or nudg* or cook* or prepar* or cater* or behav* or inten* or attitud* or social* norm*) adj7 (chang* or increas* or decreas* or improv* or reduc* or low or moderat* or shift* or eliminat* or replac* or substitut* or alternat* or cut* or more or less or free or sustainab* or promot* or encourag* or enhance* or support* or motivat* or achiev*)).ti,ab.	7008
3	((chang* or increas* or decreas* or improv* or reduc* or low or moderat* or shift* or eliminat* or replac* or substitut* or alternat* or cut* or more or less or free or sustainab* or promot* or encourag* or enhance* or support* or motivat* or achiev*) adj7 (consum* or intak* or eat* or purchas* or choos* or select* or prefer* or demand* or buy* or avoid* or choice* or nudg* or cook* or prepar* or cater* or behav* or inten* or attitud* or social* norm*) adj7 (meat* or beef or veal or lamb or pork or poultry or chicken or turkey or venison or offal* or sausage* or ham or pastrami or bacon or salami or nugget* or burger* or hamburger* or black pudding* or haggis or plant-based or vegetable-based or vegetar* or vegan* or flexitarian* or ((green or environment* or sustainab*) adj2 (eat or eating or diet*))) adj7 (consum* or intak* or eat* or purchas* or choos* or select* or prefer* or demand* or buy* or avoid* or choice* or nudg* or cook* or prepar* or cater* or behav* or inten* or attitud* or social* norm*)).ti,ab.	6734
4	1 or 2 or 3	7722
5	((randomized controlled trial or controlled clinical trial).pt. or randomized.ab. or placebo.ab. or drug therapy.fs. or randomly.ab. or trial.ab. or groups.ab.) not (exp animals/ not humans.sh.)	3647928
6	(((feasability or pilot or demonstration or multicentre* or multicentre* or multi-centre* or multi-center* or preliminary or follow-up or major) adj2 (stud* or project?)) or intervention or preintervention or postintervention or pre-post or (pre adj5 post) or quasiexperiment* or quasi-experiment* or quasirandom* or quasi-random or (before adj10 (after or during)) or ("time series" adj2 interrupt*) or ("time points" adj3 (over or multiple or day? or week? or month?))).mp. not (exp animals/ not humans.sh.)	1591076
7	5 or 6	4672088
8	4 and 7	1845
**Databases searched:**	CAB Abstracts [OvidSP](1973 to 2017 week 33), Embase [OvidSP](1974 to Aug 31, 2017), PsycINFO [OvidSP](1967 to August week 3 2017), Science Citation Index [Web of Science Core Collection)(1945 to Aug 31, 2017), MEDLINE [OvidSP](1946 to Aug 31, 2017), and Dissertations & Theses:Global full-text [Proquest] (Aug 31, 2017)	

Appendix 2: Databases search strategy

Paper	Sample characteristics and study comparison	Intervention	Outcome	Results
**Manipulation of the sensory properties of meat**				
Kunst and Hohle (2016), study 2b[1]	Sample size: N=101<br>Age: M=35, SD=11<br>Female: 60%<br>Comparison: IG vs CG, RCT	IG: Participants viewed a picture of a pork roast with the pig's head.<br>CG: Participants viewed a picture of a pork roast without the pig's head.	Attitudes towards eating the pork roast were assessed by asking participants how negative or positive they felt about eating it on a scale from 0 (extremely negative) to 100 (extremely positive).	Attitudes towards eating the pork roast were significantly less positive in the IG (M≈45, SE≈5) than in the CG (M≈64, SE≈5, t(99)=-2.77, *p*=0.007).
Kunst and Palacios Haugestad (2018), American sample[2]	Sample size: N=178<br>Age: M=36, SD=11[(a)]<br>Female: 42%[(a)]<br>Comparison: IG vs CG, RCT	IG: Participants viewed a picture of a pork roast with the pig's head.<br>CG: Participants viewed a picture of a pork roast without the pig's head.	Attitudes towards eating the pork roast were assessed by asking participants how negative or positive they felt about eating it on a scale from 0 (extremely negative) to 100 (extremely positive).	Attitudes towards eating the pork roast were significantly less positive in the IG (M≈38, SE≈3) than in the CG (M≈66, SE≈3, t(176)=-6, *p*<0.001).
Kunst and Palacios Haugestad (2018), Ecuadorian sample[2]	Sample size: N=183<br>Age: M=27, SD=9 [(b)]<br>Female: 58% (b)<br>Comparison: IG vs CG, RCT	IG: Participants viewed a picture of a pork roast with the pig's head.<br>CG: Participants viewed a picture of a pork roast without the pig's head.	Attitudes towards eating the pork roast were assessed by asking participants how negative or positive they felt about eating it on a scale from 0 (extremely negative) to 100 (extremely positive).	Attitudes towards eating the pork roast did not differ between the IG (M≈59, SE≈2) and the CG (M≈62, SE≈2, t(181)=-0.89, *p*=0.377).
**Manipulating the description of meat**				
Kunst and Hohle (2016), study 5[1]	Sample size: N=190<br>Age: M=34, SD=10<br>Female: 52%<br>Comparison: IG vs CG, RCT	IG: Food menu with 8 meat-based meals, which were described as *'cow'* and *'pig'* options.<br>CG: Food menu with 8 meat-based meals, which were described as *'beef'* and *'pork'* options.	Attitudes towards eating the meat options were assessed by asking participants how negative or positive they felt about eating them on a scale from 0 (extremely negative) to 100 (extremely positive).	Attitudes towards eating the meat options were significantly less positive in the IG (M≈51, SE≈4) than in the CG (M≈69, SE≈4) t(188)=3.59, *p*<0.001).

M=Mean, SD=Standard deviation, SE=Standard error, IG=Intervention group, CG= Control group, ≈ indicates results were read from figures or graphs. (a) Of the 201 participants enrolled. (b) Of the 202 participants enrolled.

Appendix 3: Interventions and their impact on or association with attitudes, perceived behavioural control, and subjective social norms of eating, purchasing, or selecting (less) meat.

Paper	Sample characteristics and study comparison	Outcome	Results
**Weight**			
Clark (2017)[3]	Sample size: N=37<br>Age: Median 27 (25th-95th percentile: 24-32)<br>Female: 57%<br>Comparison: Pre-post	Weight was measured using a calibrated scale before the intervention commenced and at the end of the 12 weeks intervention.	Weight did not differ significantly between the control and the intervention period (*p*=0.23).
Flynn et al (2013)[4]	Sample size: N=63<br>Age: M=52, SD=17<br>Female: 84%<br>Comparison: Pre-post	The Body Mass Index (BMI) was calculated at the baseline and 6 months post intervention using (1) non-fasted and clothed weight measured with an electronic scale with 50g gradation, whose accuracy was previously determined using standardized weights and (2) height (without shoes) measured with a tape.	BMI was marginally significantly lower 6 months post-intervention completion (M=32.9, SD=8.4) than at the baseline (M=33.3, SD= 8.5, *p*=0.05)
Holloway et al (2012)[5]	Sample size: N=25<br>Age: M=21, SD=3<br>Female: 60%<br>Comparison: Pre-post	Weight with light clothing was measured to the nearest 0.01 kg using a digital scale at the baseline and at the end of the 4 weeks intervention	The weight among males was M=74.1 (± 12.8) at baseline and 73.4 (± 10.9) at the intervention completion. The weight among females was M=59 (± 8.1) at baseline and M=59.2 (± 7.8) at the intervention completion. The author reports "no significant effects on body weight".
**Blood lipids**			
Clark (2017)[3]	Sample size: N=37<br>Age: Median 27 (25th-95th percentile: 24-32)<br>Female: 57%<br>Comparison: Pre-post	Total cholesterol, total TAG, LDL-cholesterol and HDL-cholesterol were assessed using a Pentra 400 clinical chemistry analyser on a 16 ml blood sample taken via venepuncture. Blood lipids were assessed before the intervention commenced and at the end of the 12 weeks intervention.	Total cholesterol (M=-6.8%, *p*=0.082), HDL cholesterol (*p*=0.807), and total TAG (*p*=0.864) did not decrease significantly over the intervention period, while LDL cholesterol did (M=-9.7%, *p*=0.012).
Holloway et al (2012)[5]	Sample size: N=25<br>Age: M=21, SD=3<br>Female: 60%<br>Comparison: Pre-post	Total cholesterol, HDL-cholesterol, and total TAG were measured using enzymatic colorimetric techniques with a Horiba Analyser. LDL-cholesterol was calculated using the Friedewald Equation. Analyses were performed on an 8-millilitre 12-hour overnight fasting blood sample collected by venepuncture at the baseline and at the end of the 4 weeks intervention.	Total cholesterol (baseline: 4.45±0.53, follow-up: 4.05±0.66 *p*<0.001), LDL cholesterol (baseline: 2.40±0.36, follow-up: 2.15±0.46, *p*=0.007), and total TAG (baseline: 1.14±0.53, follow-up: 0.88±0.31, *p*=0.016) were significantly lower at post intervention, while HDL cholesterol was not (baseline: 1.53±0.39, follow-up: 1.5±0.38, *p*=0.47). [(a)]
**Blood pressure**			
Clark (2017)[3]	Sample size: N=37<br>Age: Median 27 (25th-95th percentile: 24-32)<br>Female: 57%<br>Comparison: Pre-post	Systolic blood pressure (SBP) and diastolic blood pressure (DSB) were assessed at the baseline and at the end of the 12 weeks intervention.	Neither SBP (*p*=0.2) nor DBP (*p*=0.32) changed over the intervention period.

M=Mean, SD=Standard deviation, IG=Intervention group, CI=Confidence interval, OR=Odds ratio, HDL=High-density lipoprotein, LDL=Low-density lipoprotein, TAG=Triacylglyceride, SBP=systolic blood pressure, DBP=diastolic blood pressure. (a) Results were reported for thoroughness, however these data were based on an

independent sample t-test, while a dependent sample t-test is more appropriate for pre-post designs.

Appendix 4: Interventions and their impact on or association with selected biomarkers of health risk.

References

1 Kunst JR, Hohle SM. Meat eaters by dissociation: how we present, prepare and talk about meat increases willingness to eat meat by reducing empathy and disgust. *Appetite* 2016; **105:** 758–74.

2 Kunst JR, Palacios Haugestad CA. The effects of dissociation on willingness to eat meat are moderated by exposure to unprocessed meat: a cross-cultural demonstration. *Appetite* 2018; **120:** 356–66.

3 Clark M. Chronic effects of replacing red and processed meat with non/reduced meat alternatives. Unpublished.

4 Flynn MM, Reinert S, Schiff AR. A six-week cooking program of plant-based recipes improves food security, body weight, and food purchases for food pantry clients. *J Hunger Environ Nutr* 2013;**8:** 73–84.

5 Holloway T, Salter AM, McCullough FS. Dietary intervention to 1 59 reduce meat intake by 50% in University students—a pilot study. *Proc Nutr Soc* 2012; **71:** E164.
